# Energy Balance Feasibility Study for Latinas in Texas: A Qualitative Assessment

**Published:** 2007-09-15

**Authors:** Amelie G Ramirez, Patricia Chalela, Kipling Gallion, Luis F Velez

**Affiliations:** Institute for Health Promotion Research, Department of Epidemiology and Biostatistics, The University of Texas Health Science Center at San Antonio; The University of Texas Health Science Center at San Antonio, San Antonio, Texas; The University of Texas Health Science Center at San Antonio, San Antonio, Texas; The University of Texas Health Science Center at San Antonio, San Antonio, Texas

## Abstract

**Introduction:**

Obesity has reached epidemic levels, with nearly two-thirds of the U.S. population considered overweight or obese. Latinos have some of the highest rates of overweight, obesity, and sedentary lifestyle. Research from scientifically sound evidence-based interventions to reduce the disproportionate burden of obesity and its associated morbidity and mortality among Latinas is greatly needed. The purpose of this study was to assess knowledge, attitudes, and behaviors about nutrition and exercise among Latinas aged 40 years and older residing in a low-income community in Houston, Texas, and the applicability of an evidence-based church program to promote healthy energy balance.

**Methods:**

Qualitative assessment was conducted through 10 focus groups with 75 women recruited through three Catholic churches, community groups, and leaders.

**Results:**

Participants identified barriers and enabling factors to healthy nutrition and physical activity. Barriers included lack of awareness about nutrition and physical activity, cultural beliefs, and socioeconomic and environmental factors. Preferred strategies were group activities with direct guidance from qualified individuals and interpersonal contact among participants, social support with positive reinforcement for behavior change or maintenance, and a friendly environment for learning and achieving suitable goals. The church was considered a powerful resource to influence Latinas to improve their health, exercise, and nutrition practices.

**Conclusion:**

Our findings suggest that using the church environment to reach Latina women aged 40 years and older is a feasible and culturally appropriate strategy. The church environment provides a safe, comfortable, and familiar atmosphere for women and addresses specific cultural barriers and safety concerns of family members.

## Introduction

Nearly two-thirds of the U.S. population is considered overweight or obese (1), and substantial evidence suggests that excess body weight is a risk factor for many cancers, coronary heart disease, stroke, high blood pressure, and diabetes (2,3). Among Latinos, the largest minority group in the United States (41.3 million people comprising 14.1% of the total population) (4), adults have some of the highest rates of overweight (61.6%), obesity (22.6%), and sedentary lifestyle (53.2%) (5,6). Mexican American adults are especially affected. According to one study conducted between 1999 and 2002, 72.5% of Mexican American adults were overweight and obese (body mass index [BMI] ≥25.0 kg/m^2^), and 32.6% were obese (BMI ≥30.0 kg/m^2^) (7). Sex and age differences were also found within this Hispanic subgroup. Women aged 40 to 59 years had the highest prevalence of overweight and obesity among Mexican American adults, with 80.9% being overweight and obese and 47.7% being obese (7).

Obesity is considered the second largest preventable cause of death after smoking (8). The causes of obesity are multifactorial and include polygenic, metabolic, psychosocial, and environmental influences (9,10), particularly the promotion of unhealthy dietary and physical activity practices. Socioeconomic status and place of residence are other contributing factors (9,11).

Energy balance, the integrated effect of diet, physical activity, and genetics on growth and body weight over an individual's lifetime, is related to cancer risk (12), and body weight is related to cancer risk and survival. Up to 14% of all deaths from cancer may be attributable to energy imbalance (13). Results from some studies suggest that avoiding weight gain may reduce the risk for colon, breast, endometrial, kidney, gallbladder, pancreatic, and esophageal cancers (14-17). In addition to hormonal factors, other possible cancer-causing mechanisms related to overweight include gastroesophageal reflux in esophageal adenocarcinoma and increased risk of gallstones in gallbladder cancer (16,18-22). Metabolic syndrome, characterized by obesity and other health risk factors, may be related to cancer progression and response to treatment in general (23). Physical activity, an essential component of keeping caloric balance, is also important in reducing cancer risk, especially among postmenopausal women for whom gaining weight becomes an important risk factor (13). Unfortunately, overweight women are less likely than women of a healthy weight to follow cancer screening guidelines and to be thoroughly examined by their doctors (24-26).

Excess consumption of simple carbohydrates and high-fat foods, inadequate intake of dietary fiber, and reduced physical activity are associated with poor health status (27). Latinos consume fewer fruits and vegetables on a daily basis, eat more foods high in sugar and fat, and choose fast food more often when eating out compared with members of other ethnic groups (28-31).

Even when controlling for confounding socioeconomic variables, Latinos still have significantly higher BMIs than do their white counterparts (32). Although 46% of white women surveyed in the Behavioral Risk Factor Surveillance System (BRFSS) were meeting the recommendations for physical activity, only 35.6% of Latinas met the recommendations (33). Results from the National Health and Nutrition Examination Survey (NHANES) III indicated that the age-adjusted prevalence of metabolic syndrome in the United States was 23.7%, and Latina women have the highest prevalence of all groups (35.6%) (34).

Although more than 50% of women surveyed in the 2000 BRFSS were obese or overweight, 75% had not received any advice on weight loss when visiting health care professionals (35). Twenty percent of overweight or obese women thought they were at their ideal body weight, and 9.6% of obese women reported that they were in excellent health (35). More Hispanic men and women than white men and women perceive themselves as being at their ideal body weight (32).

Many positive and negative incentives to maintaining energy balance exist. Latina women have expressed their need for educational, motivational, and support groups, as well as accessible facilities and child care (33). Recent research suggests that women who observe community members exercising are more than 2.5 times as likely to meet physical activity recommendations than are women who do not observe community members exercising (33), and women who know family members who purged and used diet pills are six times as likely to mimic such behaviors than are women who do not have family members who exhibited these negative weight-loss behaviors (36). Different studies have identified specific barriers that impede the adoption of better eating practices and optimal physical activity among Latinos. Examples of these barriers include income and education; cultural and family patterns; attitudes and beliefs; time constraints; personal choices; lack of social support; television viewing; and environmental factors, such as poor street and sidewalk maintenance, personal safety concerns, and lack of exercise resources (37-44). Sociodemographic variables associated with obesity and trying to lose weight vary by race and ethnicity, indicating the need to develop tailored models specific to Latinos for effective responses to weight management (35).

This paper presents the findings from 10 focus groups conducted to assess knowledge, attitudes, and behaviors about nutrition and exercise among Hispanic women aged 40 years and older residing in a low-income community in Houston, Texas, and the applicability of an evidence-based church program to promote healthy energy balance. The findings will guide the design of a culturally sensitive program, tailored to the needs and preferences of this specific group.

## Methods

### Study setting

From May through June of 2005, 10 focus groups were conducted at three Catholic churches located in the East End district of Houston, Texas, a large, predominantly Hispanic (85.6%) area (4). The majority of Hispanics are Catholic (70%), and more than 90% of Mexican American women frequently attend church and participate in church events and committees (45,46). The district's population is 136,072, and approximately 20% of women are aged 40 years and older (4). The median family income range in 2000 was between $21,449 and $38,214, but a large proportion of the community (27.4%) has an income below $20,000. More than 68.7% of the population never completed high school, and 88.2% speak Spanish at home. Approximately 48% of the population are foreign-born, and 53.2% have lived in the same house for 5 years or more (4).

### Participants and recruitment

Using convenience and snowball sampling methods, we recruited 75 low-income women to participate in the focus group sessions. Eligibility criteria included 1) being a woman 40 years of age or older, 2) being Hispanic, and 3) residing in the East End district of Houston, Texas.

A master agreement was established with three local churches to coordinate the recruitment of study participants and to provide logistical support for focus groups, including the use of church facilities to conduct the groups. The churches assisted in the identification of active community leaders who in turn provided information that enhanced the recruitment of women from other community groups and organizations. A promotional flyer featuring general study information was distributed in each church's weekly bulletin. Project staff gave presentations to different community groups (e.g., senior centers). Eligible women provided their names and phone numbers to community leaders, who in turn gave the list of potential participants to the secretary of each church. Once identified, potential participants received a call from the church secretary one week before the meeting to confirm their planned attendance and then one final reminder phone call a day before the meeting. The study's community coordinator maintained close contact with church leaders throughout the recruitment period. Focus groups were held at participating churches at the most convenient time for participants, usually in the evening. All participants were compensated $25.00 for their participation in the study and signed a consent form before participating.

### Interview guide

With the purpose of exploring knowledge, attitudes, and practices related to general health, excess weight, nutrition, physical activity, and community resources, study researchers developed interview guides based on literature reviews and the team's own experience. Questions were translated into Spanish by a bilingual research team member and reviewed by the entire team. In addition, as requested by the Special Populations Network for Cancer Control (managed by the Health Disparities Research branch of the Center to Reduce Cancer Health Disparities), 16 strategies drawn from the physical activity section of the *Guide to Community Preventive Services* were included to determine their appropriateness and cultural relevance on the basis of perceived benefits and barriers identified by participants (47).

### Focus group procedures and data collection

The Baylor College of Medicine Institutional Review Board approved the English and Spanish interview guides and consent forms, including the authorization to audiotape. The focus group discussions were moderated by a trained, bilingual Hispanic member of the project staff. Following the preferences of the groups, one session was conducted in English and in Spanish and the other sessions in Spanish only. A second bilingual member of the research team observed the group interactions, took field notes, operated the tape recorder, and provided logistic support for each session. The sessions began with introductions, a brief explanation of the purpose of the meeting, and general ground rules for the session. The consent form was read aloud by the moderator, and questions were answered to clarify any doubts. All participants signed the consent form before the discussion started. Demographic information was collected using a brief five-item questionnaire. Focus groups lasted on average 1.5 hours. All focus groups were audio recorded and transcribed verbatim.

### Data analysis

Focus group data were analyzed using a general inductive coding approach (48)Focus group data were analyzed using a general inductive coding approach (48). Research team members read and analyzed the transcripts independently. The analysis included 1) reviewing the transcripts thoroughly; 2) openly coding each response, using the interview categories as a guide; 3) writing impressions and labeling segments of text with coding categories; 4) reviewing and refining categories; 5) identifying recurrent and additional emerging themes; and 6) deriving final themes. The research team met several times to compare and discuss all theme categories for validation, and minor disagreements were reviewed and discussed until consensus was achieved. Salient quotations were extracted from the transcripts and included in the final report to underscore different responses or conclusions. Demographic data were analyzed using SPSS software, version 14.0 (SPSS, Inc, Chicago, Illinois).

## Results

Ten focus groups were conducted with an average of seven participants per group (sizes ranged from 5 to 12 participants). Demographic characteristics are presented in Table 1. The mean age was 56 years (SD = 10.6 years), and ages of participants ranged from 40 to 80 years. About 61% of participants were married, and 23% had only a high school education. Most participants (64%) reported a yearly family income of less than $20,000, with only a small number of participants (9%) reporting a yearly income of more than $40,000.

Table 2 lists the major themes that were identified. General knowledge and perceptions about health included discussions about primary diseases in the community, their risk factors, and preventive and control measures. Themes for nutrition and physical activity were related either to barriers or to motivators and enabling factors (e.g., awareness, culture, beliefs and attitudes, environmental and socioeconomic factors) for eating healthfully and performing exercise and involved discussions about church involvement in promoting healthy eating and exercise habits in the community. Additional themes for physical activity included preferred activities by participants and assessment of strategies from the *Guide to Community Preventive Services* to promote physical activity in the community (47). Preferred means of receiving information on nutrition and exercise was also identified as a theme.

### General knowledge and perceptions about health

Diabetes, high blood pressure, and cholesterol were the major health problems identified by participants. Other less frequently cited health problems were stress, allergies, cancer, use of alcohol and drugs, and depression. The most frequent risk factors mentioned by participants were stress, eating habits, heredity, and environmental pollution. When prompted, participants agreed that overweight and obesity were frequent health problems in their community.

### Barriers to eating healthfully

Top barriers keeping Latina women from eating healthfully included lack of awareness and knowledge about all available vegetables and how to cook them, the nutritional value of foods, food combinations and portion sizes, reading food labels, and what to buy on a limited budget. Women considered that beliefs related to traditional food preparation and selection and the perception that being "chubby" or overweight is healthy are important influencing factors.

Participants reported that because of budget limitations they prefer foods that are on sale and provide a feeling of fullness. They indicated that vegetables and fruits are usually expensive and are not their first choice when buying groceries. Family preferences were also considered a barrier to eating healthfully. Many women work and lack the time to prepare meals, making it easier to buy convenient or prepackaged food. In addition, women who have to work lack a regular schedule to eat meals. Many participants do not have time to eat breakfast and often skip lunch, instead just having a snack and a big, heavy, and usually unhealthy meal in the evening.

### Enabling factors and motivators to eating healthfully

Top enabling and motivating factors discussed by participants included addressing the barriers to eating healthfully. Most women expressed a strong desire for cooking classes conducted in small groups that provide samples to take home for tasting, education about how to buy and cook healthy traditional foods on a limited budget, and education on the relationship between nutrition and health and diseases. Participants reported that education provided through small groups at the church would provide a familiar environment and address additional barriers to attending classes (e.g., the influence of *machismo* on women*,* social support, safety issues).

### Barriers to performing physical activity 

Top barriers keeping Latinas from being more physically active included safety concerns and fear of going out to exercise, very limited community resources available for physical activity and exercise, cost of existing programs and resources, lack of time and motivation (i.e., *flojera*), cultural factors such as *machismo* (i.e., spouses/partners do not want them to go out and exercise with other men), modesty (i.e., feeling embarrassed by how they look in gym attire), familism (i.e., putting family needs before their own), and not wanting to miss *novelas* (i.e., soap operas). (Watching *novelas* on Spanish-language television is a common leisure practice and a deeply rooted cultural tradition among Latinos.) Women also mentioned lack of social and family support, lack of education and information about the benefits of exercising, and lack of education and information about appropriate types of exercises according to age, fitness level, and health as important barriers.

### Enabling factors and motivators to performing physical activity

Top enabling factors and motivators to being more physically active and exercising regularly included addressing the barriers to performing physical activity and being provided with small-group–style education about the benefits of exercise; types of exercise based on age, fitness level, and health; and how to begin and maintain an exercise program. Participants liked small groups because members could encourage and support one another and provide positive reinforcement. The women indicated that programs should address cultural factors and be offered at convenient times. Participants reported that programs through the church would provide a safe, convenient, and familiar environment and be easily accepted by family, especially the spouse. In order of importance, walking, dancing, aerobics, swimming, bicycling, and yoga were the preferred physical activities mentioned by women participating in the focus groups.

### Church involvement

Participants identified several ways churches could help to improve Latinas' nutrition and exercise practices. Ideas included providing space for cooking, nutrition, and exercise classes; creating groups for nutrition and exercise; providing classes (e.g., healthy eating and food preparation, dancing, walking, aerobics, yoga) conducted by people who know about the topic; and having the priest participate actively (i.e., participating in groups to promote healthy eating and exercise).

### Information preferences

Doctors, nurses, and health care professionals were mentioned as the most common and trusted sources of health information for Latinas in this community. Other trusted sources of information included priests, television, libraries, the Internet, and books. All participants reported that information and education on nutrition and exercise should be provided in person, specifically through group sessions or activities. The materials and messages should preferably be in Spanish and English.

### Physical activity strategies assessed by focus group participants

Table 3 shows a summary of the strategy assessment by focus group participants. Sixteen strategies were assessed by participants. Each strategy was read by the moderator, and each woman provided her opinion about perceived benefits and barriers. On the basis of this discussion, participants voted for or against the specific strategy. The three strategies that participants preferred included conducting community-wide campaigns, providing social support in community settings, and creating or enhancing access to places for physical activity.

Within the preferred strategy of conducting community-wide campaigns, health fairs were the only activity selected, because they involve a group or community activity, are free, respond to a specific health need, and provide an immediate outcome (e.g., seeing a doctor, getting an exam).

Within the preferred strategy of providing social support in community settings, the specific activities selected included having a *comadre* (i.e., "buddy") who goes to exercise classes and meetings with you, joining an exercise group (e.g., walking group), and taking part in small-group discussions about health with a counselor. Small-group discussions included participating in group exercises, setting a walking goal and adhering to it (group effort), and having your name placed in a lottery for a monthly prize. Benefits of these activities include the encouragement, support, and reinforcement of group activities; social interaction; the opportunity to learn and practice at the same time; and the incentive for participating. Not having child care was considered the main barrier to this strategy, followed by the need for having group participants with similar fitness levels.

Within the preferred strategy of creating or enhancing access to places for physical activity, participants preferred using outdoor trails for walking and running and/or using exercise rooms and equipment for aerobics classes and weight conditioning. Women were in favor of these activities if they were conducted as a group or involved the family. Major barriers to this strategy included safety issues, lack of resources, and lack of appeal if women have to exercise by themselves. Participants preferred going to a gym that does not charge a membership fee and has convenient hours. Additional barriers to this strategy were transportation and cultural issues (e.g., modesty, *machismo*). Women expressed a desire to have a gym that is only for women, so they do not feel embarrassed by how they look in gym attire and so spouses do not become jealous.

Most participants were opposed to individual behavior-change strategies, activities involving reading, and activities that required financial expenditure. These types of activities elicited the most barriers for implementation and were not appealing to participants. Six activities to promote physical activity related to community-wide campaigns, individual behavior change programs, and point-of-decision prompts to encourage stair use were not viewed favorably by any of the women. Within the community-wide campaign strategy, obtaining a packet of information with tips on how to move more and going to a class and watching a video to learn ways to move more were activities rejected by participants. Main barriers to this strategy included literacy level, lack of time for reading, and lack of appeal and motivation.

The least appealing individual behavior change strategies included 1) working with a personal trainer at home, 2) working with a fitness counselor who would help find ways to change the things that keep participants from exercising, 3) keeping track of the number of steps taken each day using a step counter, and 4) using a device to measure heart rate. Most participants said they do not like working out alone.

Within the strategy of point-of-decision prompts to encourage stair use, women were against printed messages or signs by the elevator to encourage them to use the stairs instead of the elevator or escalator or to park farther away to walk more. Main barriers included lack of time, safety and health issues, literacy level, and lack of appeal. Most women reported that these signs would not motivate them to act.

## Discussion

The study findings are consistent with other studies that found that poor association between health and weight, lack of awareness about nutrition and physical activity, cultural factors and beliefs, poverty, lack of time, lack of social and family support, safety concerns, and lack of community resources were important barriers that keep Latinas from eating healthfully and being more physically active (9,33,37-44,49-52).

Family and social support networks are very powerful within the Hispanic culture (49,51,53). As expected, and in agreement with previous studies (49,50), most Hispanic women preferred strategies that involve group activities with direct guidance from qualified individuals and interpersonal contact among participants, social support with positive reinforcement for behavior change and maintenance, and a friendly environment for learning and achieving suitable goals.

Most participants were opposed to individual behavior change strategies, activities involving reading, and those that required financial expenditure. These types of activities elicited the most barriers for implementation and were not appealing to participants. Given the literacy level of members of this group and the fact that they generally do not like to read, printed materials, if any, might be more useful as a complement to group activities and should be attractive, with short, easy-to-understand messages.

Previous research has suggested that churches play an important role in achieving effective results for social change and health promotion behaviors among Latinos (51,53-55), and support from church staff may represent a powerful motivator to participate (51). The church may be a suitable community setting for implementing programs that provide women with the knowledge, skills, and motivation to become more active (33) and eat more healthfully and, consequently, to maintain a healthy weight. Consistently, our results suggest that using the church environment to reach Latina women aged 40 years and older is a feasible and culturally appropriate strategy. The church provides a safe, comfortable, and familiar atmosphere for women and addresses safety concerns of family members.

A very important barrier to healthy eating found in this study is inappropriate distribution of meals throughout the day because of lack of time. Women do not have time to eat a healthy breakfast in the morning and often skip lunch. Instead, they tend to pack most of their calories into the evening meal. We think this is an important reason for concern that warrants further study. Skipping breakfast and eating large dinners affects the way the tissues respond to the influence of insulin and glucagon, ultimately facilitating resistance, a key problem in the development of metabolic syndrome and other obesity-related problems. We believe that this pattern may be affecting Latinos of all ages. Since Latino families tend to have larger numbers of children and since women are responsible for meal preparation in these families, our observations suggest that children also may often be skipping breakfast and experiencing heavy caloric consumption during the school lunch and later in the day.

In the area of behavioral research, well-designed, long-term clinical trials are needed to evaluate various methods and strategies for voluntary weight loss among minority populations. Research on the prevention of overweight and obesity and unhealthy weight gain among low-income Latinas is of critical importance. Voluntary weight-loss practices are closely related to cultural factors and societal attitudes toward weight and body image. Interdisciplinary research is necessary to develop and evaluate prevention and control programs that encourage Latinas to adopt and maintain healthy eating habits and lifestyles for lifelong control of weight.

Several limitations should be noted. The qualitative design of this study does not allow for causal inferences or generalizations, so results must be interpreted with caution. Similarly, the sampling methods provided a self-selected and fairly homogenous group of low-income Latinas aged 40 years and older who may not be representative of the entire target population. Consequently, the findings cannot be generalized to women of other ethnic minority groups, higher socioeconomic status, or younger age, or to women who do not attend church, are not closely connected with a church, or who live in rural areas. It has been shown that, compared with residents in safer neighborhoods, residents of poor and unsafe neighborhoods are significantly less active due to limited opportunities to participate in regular physical activity and are less likely to have access to healthy foods (9,38,40,43,44,52). Finally, life experiences related to health and personal perceptions may be different for women who live in poverty and environments with limited resources than for women living with more financial stability.

Despite its limitations, this study provides important information on low-income Latinas' perceptions, needs, barriers, enabling factors, and preferences related to nutrition and physical activity for the design and implementation of culturally sensitive and tailored church-based programs. To ensure success, women and community leaders should be involved in all phases of the program to develop trust and mutual acceptance. A community-based participatory research approach is more likely to produce meaningful changes in the community, empowering Latinas to take control of their own health experiences and advocate for policy and environmental changes to enhance program sustainability.

Prevention is the most feasible approach to countering overweight and obesity. The cost of treating and managing the disabilities and diseases caused by overweight and obesity imposes an economic and health burden on society and especially on poor communities. Local and state governments, in partnership with all relevant stakeholders, urgently need to integrate strategies that promote healthy diets and regular physical activity in relevant policies and programs. Policy changes to promote physical activity will have a long-term impact on obesity-related morbidity and mortality and specifically on chronic diseases, such as cancer, diabetes, and heart disease. The causes of overweight and obesity are multifactorial, and to address this problem effectively, more research is needed on the roles of genes, metabolism, diet, physical activity, and the influence of the social and cultural environment.

## Figures and Tables

**Table 1:** Demographic Characteristics of Focus Group Participants (N = 74^a^), East End District, Houston, Texas, 2005

Characteristic	No. of Participants (%)
**Age, y **	
40-49	20 (27.0)
50-59	23 (31.1)
60-69	20 (27.0)
70-79	10 (13.5)
≥80	1 (1.4)
**Sex**	
Female	74 (100.0)
Male	0 (0)
**Ethnicity**	
Hispanic	74 (100.0)
**Country of origin**	
Mexico	69 (93.2)
El Salvador	3 (4.1)
Costa Rica	1 (1.4)
Honduras	1 (1.4)
**Marital status**	
Married	45 (60.8)
Widowed	11 (14.9)
Single	8 (10.8)
Divorced	6 (8.1)
Separated	4 (5.4)
**Educational level**	
<High school graduate	48 (64.9)
High school graduate	17 (23.0)
>High school graduate	9 (12.2)
**Family income^b^, $**	
<20,000	45 (64.3)
20,000-40,000	19 (27.1)
>40,000	6 (8.6)

a Total number does not equal 75, because one participant refused to answer demographic questions.

b Data on four participants are missing in this category because of nonresponse.

**Table 2:** Themes and Sample Comments From Focus Group Participants (N = 75), East End District, Houston, Texas, 2005

Themes	Sample Comments
General knowledge and perceptions about health	"The main health problems here are diabetes, high blood pressure, and cholesterol. . . ." ". . . [The main] causes are stress and heredity."
Barriers to eating healthfully	"Budget . . .we have to buy things that are on sale because we do not have enough money." ". . . A chubby child is healthy . . . '*está gordito, saludable*' . . . if the child is thin you think that he must be sick. . . ." ". . . Same thing with a woman just married, if she looks *gordita*, we say, marriage is going well for her. . . ." ". . . Nowadays we do not have time . . . I have to work and don't have time to eat breakfast or cook." "We do not know how to prepare a healthy meal on a limited budget. . . ." "Food doesn't taste the same without lard . . . my family doesn't like it."
Enabling factors and motivators to eating healthfully	". . . More education about how to prepare nutritious foods on a limited budget. . . ." "How to cook traditional meals without the fat but with good taste. . . ."
Barriers to performing physical activity	"Lack of motivation . . . interest . . . what we called *flojera.* . . ." "Lack of time . . .you need to take care of your family. . . ." "We do not feel safe to go out and exercise . . . I used to go to the park with my friends but since a robbery happened . . . I'm afraid." "I think [husbands'] jealousy is a problem. . . ." "*Novelas* . . . we don't want to exercise because we don't want to miss *la novela*. . . ."
Enabling factors and motivators to performing physical activity	"We need more information about what exercises are good for us . . . for our age. . . ." "Having group activities . . . alone no . . . it won't work . . . because we would support each other."
Preferred physical activities	"I love to dance . . . I dance by myself . . ." "Yes, dancing classes . . . it feels good . . . it relaxes you. . . ." "Just walking with others . . . with your *comadres*. . . ."
Assessment of NCI's proposed strategies to promote physical activity (best strategies selected)	"Health fairs are good because they are a community event . . . you receive free check-ups and you can take your family with you. . . ." "Group activities are the best . . . you can go walking with your *comadre* . . . you support each other." "Having free or low-cost gyms for women like us. . . ."
Church involvement	"Cooking classes or an exercise program offered here at the church . . . that is a good idea . . . I would feel with more motivation [*sic*]. . . ." "If the priest invites us to walk with him. . . ." "My husband wouldn't mind if is at the church. . . ."
Information preferences	"In meetings . . . with other people. . . ." "In person . . . like this *platica* . . . you hear the opinion of others and is in a group. . . ." "In Spanish . . . most people speak Spanish. . . ." "But many women speak only English. . . ."

**Table 3:** Assessment of Strategies^a^ to Promote Physical Activity by Focus Group Participants (N = 75)**,** East End District, Houston, Texas, 2005

Strategies	Perceived Benefits and Barriers	
Community-wide campaigns: Receive a pack of information by mail Go to class and watch a video Go to a health fair	Perceived benefits	Only health fairs were selected, because they involve a group/community activity, are free, respond to a specific health need, and provide an immediate outcome (i.e., seeing a doctor or getting an exam).
	Perceived barriers	Women do not like activities such as getting a packet of information and going to a class to watch a video. Main barriers were lack of appeal and motivation, literacy level, and lack of time for reading. Activities that involve interaction with others are preferred.
Individual behavior change programs: Work with a personal trainer Work with a fitness counselor Keep track of the number of steps taken	Perceived benefits	These will work only for a few people who like individual activities but not for the majority.
	Perceived barriers	Lack of appeal and motivation of individual activities, cultural issues (e.g., *machismo*), lack of time. Women prefer group activities and do not feel that individual approaches will work. There is no motivation for working out alone. Most participants were opposed to individual behavior change strategies, activities involving reading, and financial expenditure. These types of activities elicited the most barriers for implementation and were not appealing to participants.
Social support in community settings: Have a "buddy" who goes to exercise and to classes with you Join an exercise group Take part in small-group discussions with a counselor Join a group to reach a walking goal Compete with another team Receive lottery prizes for exercising	Perceived benefits	Most women preferred strategies that involve group activities with direct guidance, interpersonal contact, and social support, allowing participants to encourage and support one other and providing positive reinforcement for behavior change and/or maintenance. This strategy also provides a friendly environment for learning and for achievement of suitable goals.
	Perceived barriers	Lack of child care and need for having group participants with similar fitness levels, so women will not become discouraged.
Creating or enhancing access to places for physical activity: Use outdoor trails and gym facilities Go to gym at convenient hours Go to a free gym	Perceived benefits	In general, women were in favor of this strategy if they are in groups or involve the family. Benefits: the possibility of using the facilities free of charge and the flexible hours.
	Perceived barriers	Safety issues, lack of resources, transportation, and lack of appeal if they have to work out alone. Other barriers were cultural in nature, including beliefs such as modesty and *machismo*. Women would like to have a gym that is only for women, so they do not feel embarrassed by how they look in gym attire and spouses won't complain because of jealousy issues.
Point-of-decision prompts to encourage stair use: Use the stairs instead of the elevator Park further away to walk more	Perceived benefits	None
	Perceived barriers	There are no elevators or escalators in the community. Lack of time, safety and health issues, literacy level, and lack of appeal.

a Sixteen strategies drawn from the physical activity section of the *Guide to Community Preventive Services* (47).
